# Continued Emissions of the Ozone-Depleting Substance Carbon Tetrachloride From Eastern Asia

**DOI:** 10.1029/2018gl079500

**Published:** 2018-09-28

**Authors:** M. F. Lunt, S. Park, S. Li, S. Henne, A. J. Manning, A. L. Ganesan, I. J. Simpson, D. R. Blake, Q. Liang, S. O’Doherty, C. M. Harth, J. Mühle, P. K. Salameh, R. F. Weiss, P. B. Krummel, P. J. Fraser, R. G. Prinn, S. Reimann, M. Rigby

**Affiliations:** 1 School of Chemistry, University of Bristol, Bristol, UK; 2 Kyungpook Institute of Oceanography, College of Natural Sciences, Kyungpook National University, Daegu, South Korea; 3 Department of Oceanography, School of Earth System Sciences, Kyungpook National University, Daegu, South Korea; 4 Empa, Swiss Federal Laboratories for Materials Science and Technology, Dübendorf, Switzerland; 5 Hadley Centre, UK Met Office, Exeter, UK; 6 School of Geographical Sciences, University of Bristol, Bristol, UK; 7 Department of Chemistry, University of California, Irvine, CA, USA; 8 Atmospheric Chemistry and Dynamics, NASA Goddard Space Flight Center, Greenbelt, MD, USA; 9 Scripps Institution of Oceanography, University of California, San Diego, La Jolla, CA, USA; 10 Climate Science Centre, CSIRO Oceans and Atmosphere, Aspendale, Victoria, Australia; 11 Center for Global Change Science, Massachusetts Institute of Technology, Cambridge, MA, USA

## Abstract

Carbon tetrachloride (CCl_4_) is an ozone-depleting substance, accounting for about 10% of the chlorine in the troposphere. Under the terms of the Montreal Protocol, its production for dispersive uses was banned from 2010. In this work we show that, despite the controls on production being introduced, CCl_4_ emissions from the eastern part of China did not decline between 2009 and 2016. This finding is in contrast to a recent bottom-up estimate, which predicted a significant decrease in emissions after the introduction of production controls. We find eastern Asian emissions of CCl_4_ to be 16 (9–24) Gg/year on average between 2009 and 2016, with the primary source regions being in eastern China. The spatial distribution of emissions that we derive suggests that the source distribution of CCl_4_ in China changed during the 8-year study period, indicating a new source or sources of emissions from China’s Shandong province after 2012.

## Plain Language Summary

Carbon tetrachloride is one of several man-made gases that contribute to the depletion of the ozone layer high in the atmosphere. Because of this, restrictions were introduced on the use of this ozone-depleting substance, with the expectation that production should by now be close to 0. However, the slower than expected rate of decline of carbon tetrachloride in the atmosphere shows this is not the case, and a large portion of global emissions are unaccounted for. In this study we use atmospheric measurements of carbon tetrachloride from a site in East Asia to identify the magnitude and location of emissions from this region between 2009 and 2016. We find that there are significant ongoing emissions from eastern China and that these account for a large part of the missing emissions from global estimates. The presence of continued sources of this important ozone-depleting substance indicates that more could be done to speed up the recovery of the ozone layer.

## 1. Introduction

Carbon tetrachloride (CCl_4_) was historically used as a solvent, cleaning agent, and as a feedstock in the production of other compounds such as the chlorofluorocarbons (CFCs; Carpenter et al., 2014). While CCl_4_ production and consumption for dispersive applications has been banned under the Montreal Protocol since 2010, it continues to be produced for certain permitted exemptions and for nondispersive feedstock applications. These feedstock uses are unregulated since it is assumed that nearly all CCl_4_ produced is subsequently used, recycled, or destroyed.

Based on levels of feedstock production reported to the United Nations Environment Programme, previous studies estimated that global emissions should be less than around 5 Gg/year (Montzka et al., 2010). However, several studies have noted that the decline of CCl_4_ in the background atmosphere has progressed much more slowly than is implied by this emission rate and our understanding of atmospheric sinks (Carpenter et al., 2014; Liang et al., 2014; Montzka et al., 2010; SPARC, 2016). This inconsistency has led to a renewed focus on both the estimation of global emissions and total atmospheric lifetime of CCl_4_ (Liang et al., 2014; SPARC, 2016).

Recently, the lifetime has been revised from 26 to 33 years, based primarily on a revision of the lifetime with respect to oceanic and soil losses (Butler et al., 2016; Rhew & Happell, 2016; SPARC, 2016). As a result of this longer lifetime estimate, the global emissions estimate based on atmospheric data was lowered from 57 to 35 Gg/year for 2014 (SPARC, 2016). To address the gap between the global estimate inferred from atmospheric data and near-zero emissions based on reported production and feedstock usage, new sources of CCl_4_ have also been proposed, such as inadvertent emissions from chlor-alkali plants, and nonfeedstock emissions from the production of chloromethanes (CMs) or perchloroethylene (PCE; Bie et al., 2017; Fraser et al., 2014; Sherry et al., 2017; SPARC, 2016). It is thought that these sources could contribute up to 25 Gg/year to the global budget.

In contrast to global emissions estimation using atmospheric data and models, regional inverse modeling studies are relatively insensitive to uncertainty in the CCl_4_ lifetime and can help to address the geographical source of the discrepancy between emission estimates derived from United Nations Environment Programme production and feedstock reports, and the observed global mole fraction derived estimates. There have been a limited number of regional inverse modeling studies of CCl_4_ emissions (where measurements of CCl_4_ are used in conjunction with a transport model and statistical inference method to estimate emissions) covering some of the major economically active areas of the world. Emissions from the United States (4.0 Gg/year during 2008–2012; Hu et al., 2016), Western Europe (2.2 Gg/year during 2006–2014; Graziosi et al., 2016), and Australia (0.17 Gg/year between 2004 and 2011; Fraser et al., 2014) have all recently been estimated. The combination of these regional emission estimates is still around 30 Gg/year smaller than the estimated global emissions derived from atmospheric data.

In the 2000s, studies using atmospheric data showed that eastern Asia could contribute up to half of the global CCl_4_ emissions (Palmer et al., 2003; Vollmer et al., 2009; Xiao et al., 2010). Recent emission estimates based on CCl_4_ consumption and production data in China suggest that this contribution has fallen dramatically, particularly after 2010 (Bie et al., 2017). There have been a few recent studies that have used interspecies correlations (ISCs) of CCl_4_ atmospheric mole fractions to carbon monoxide (CO) and HCFC-22 (CHClF_2_) to infer emissions of CCl_4_ (Bie et al., 2017; Park et al., 2018; Wang et al., 2014). This method requires that the spatial distribution of CCl_4_ emissions is similar to that of emissions of the correlating species (i.e., in the case that CO or HCFC-22 are used, it assumes that CCl_4_ emissions are largely population weighted). However, the emissions estimates from the ISC methods may be prone to error in this assumption, and the two most recent studies estimate very different emission magnitudes of under 5 Gg/year (Bie et al., 2017) and 23.6 ± 7.1 Gg/year (Park et al., 2018) between 2011 and 2015. Therefore, the use of an independent inverse modelling method that is not subject to the same potential biases is important to evaluate which of these scenarios is most likely.

In this work, we follow an inverse modeling approach, where 8 years of near-continuous CCl_4_ data are used from the Advanced Global Atmospheric Gases Experiment (AGAGE; Prinn et al., 2018) station at Gosan, South Korea. These data are used in conjunction with two atmospheric transport models, the Numerical Atmospheric dispersion Modelling Environment (NAME; Jones et al., 2007; Manning et al., 2011) and the FLEXible PARTicle dispersion model (FLEXPART; Stohl et al., 2005), and two different statistical methods to investigate the magnitude and spatial distribution of emissions from the region of eastern Asia surrounding Gosan between 2009 and 2016. Significant changes in emissions may be expected during this period, given the Montreal Protocol phaseout schedule.

## 2. Trends in Atmospheric CCl_4_ Data

**Figure 1.**
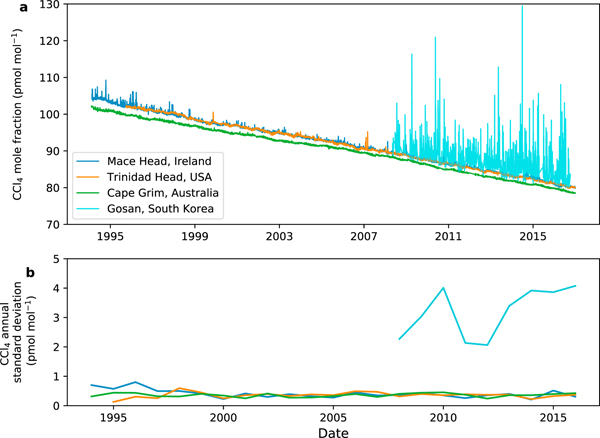
(a) Daily mean measurements of CCl_4_ mole fractions at four AGAGE stations in Europe, North America, Australia, and East Asia. Southern Hemisphere mole fractions are lower than those in the Northern Hemisphere due to stronger emissions in the latter and a 1- to 2-year interhemispheric mixing timescale. Pollution episodes are most prevalent at Gosan in South Korea. (b) Standard deviation of daily averaged CCl_4_ mole fractions in each year at the same four AGAGE stations. AGAGE = Advanced Global Atmospheric Gases Experiment.

The presence of emissions from eastern Asia can be seen in the measured mole fraction data of CCl_4_ from selected sites of the AGAGE network (Figure 1a). These data, plotted as daily mean values (but sampled at a maximum frequency of approximately 2-hourly), show how the global background levels of CCl_4_ have decreased since the early 1990s as a result of the drop in global emissions affected by the mandates of the Montreal Protocol and subsequent amendments. Some enhancements above baseline levels are visible in Figure 1a in the 1990s at the European Mace Head station, with the standard deviations at this site (shown in Figure 1b) slightly greater than at Cape Grim, Australia, before converging to very similar values, representing mostly baseline variations, by the year 2000.The latter part of the measurement record is dominated by mole fraction enhancements from the Gosan station in South Korea, where 2-hourly measurements have been available since 2008. Gosan is located on Jeju Island, 100 km south of the Korean Peninsula and 500 km north east of Shanghai, China, with a prevailing wind direction from the northwest (Li et al., 2011). Thus, the large enhancements above baseline levels at this site are likely to be indicative of significant sources of emissions in eastern Asia. Although there is some interannual variability, the magnitude and frequency of the peaks at Gosan have not shown a consistent decrease since 2008 (Figure 1b), implying that there has not been a significant decrease in emissions during this period from the region (assuming no changes in atmospheric transport). For the purposes of this work we define the eastern Asia emissions region to be bounded by 18°N to 50°N and 104°E to 147°E (see Figure 3). Although a significant proportion of China lies west of this region, the measurements have little sensitivity to emissions from outside this region, and hence, we have limited our analysis to this smaller domain surrounding the Gosan measurement site.

## 3. Eastern Asia Emissions 2009–2016

**Figure 2.**
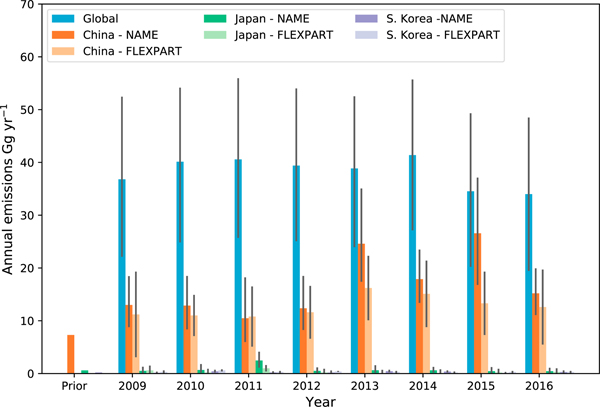
Global (blue) and eastern Asia emissions of CCl_4_ between 2009 and 2016, showing China (orange), Japan (green), and South Korea (purple). Eastern Asia emissions were estimated using 2-hourly data from Gosan, South Korea, with darker shades of each color representing emissions estimated using NAME, and lighter shades using FLEXPART. Bottom-up industrial estimates from Sherry et al. (2017) used as the prior in our regional inversions are also shown. Global emissions were derived using monthly background data at Advanced Global Atmospheric Gases Experiment sites and a 2-D box model (Rigby et al., 2014). The black bars represent the 90% confidence range of the annual estimates from NAME and 2*σ* standard deviations from the mean for the FLEXPART and global estimates. NAME = Numerical Atmospheric dispersion Modelling Environment; FLEXPART = FLEXible PARTicle dispersion model.

The NAME and FLEXPART models were used to predict the sensitivity of mole fraction measurements at Gosan to emissions from the model grid cells. For each model a separate Bayesian inverse modeling approach was used to infer emissions (Henne et al., 2016; Lunt et al., 2016), further details of which are in the supporting information Text S2. Figure 2 shows our estimates of annual mean emissions from China, Japan, and South Korea for the period 2009–2016 derived using high-frequency data from Gosan. We find emissions from China to be the dominant source of CCl_4_ in this region, averaging 17 (11–24) or 13 (7–19) Gg/year for the NAME and FLEXPART estimates, respectively, over this 8-year period. Our estimates for China are significantly higher than the recently published bottom-up estimates of 4 Gg/year (Bie et al., 2017) and 7 Gg/year (Sherry et al., 2017) and suggest unaccounted for sources in the bottom-up estimates. For comparison, estimates of global emissions using monthly background data from AGAGE sites and calculated using a 2-D box model (Rigby et al., 2014) are also shown in Figure 2. These top-down global emissions are an update of previously reported estimates (SPARC, 2016) and show relatively steady emissions averaging 38 (24–53) Gg/year during this 8-year period. Our emissions estimates from countries in the eastern Asia region also show no persistent overall trend, in contrast to the previous finding that emissions from China declined significantly after 2010 (Bie et al., 2017), although the NAME estimates for China exhibit some large interannual differences between 2012 and 2016.

**Figure 3.**
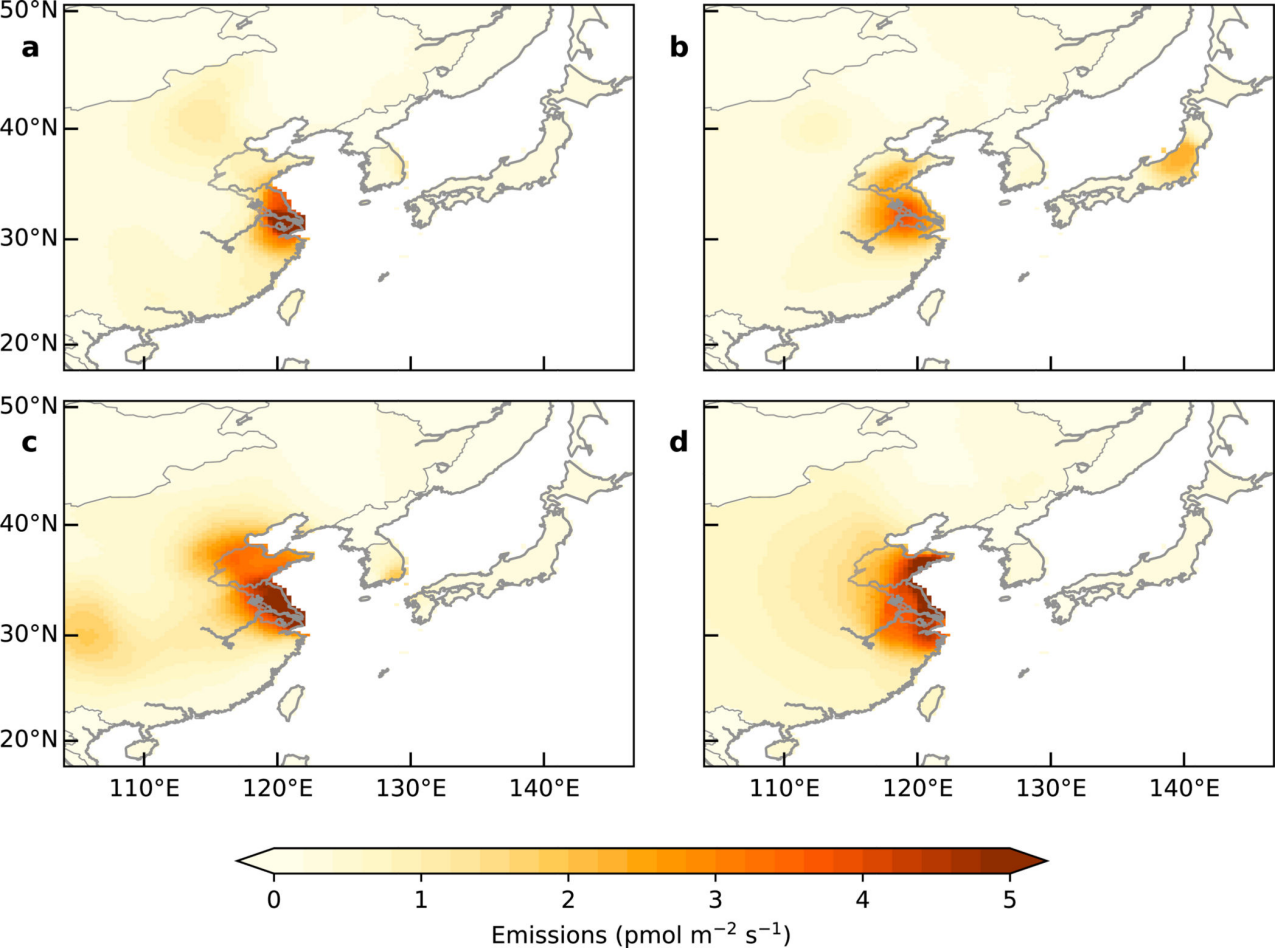
Mean spatial distribution of posterior emissions from Numerical Atmospheric dispersion Modelling Environment inversions during (a) 2009–2010, (b) 2011–2012, (c) 2013–2014, and (d) 2015–2016. Darker colors represent regions of highest emissions, which are concentrated in eastern China. The borders of Jiangsu and Shandong provinces in China are outlined in gray.

The distribution of emissions derived using NAME indicates that sources of CCl_4_ are predominantly from the eastern provinces of China, around Jiangsu, Shanghai, and Shandong (Figure 3). A similar distribution is also seen in the posterior estimates from FLEXPART (see supporting information Figures S9 and S10). These provinces include major industrialized areas such as the Yangtze River Delta and have previously been identified as the source region of another chlorinated methane (CM), methyl chloride (CH_3_Cl; Li et al., 2016). In China and elsewhere, CCl_4_ is a by-product of CM and PCE production and is used in the production of CM, PCE, hydrofluorocarbons, and divinyl acid chloride, which all have the potential for unintended emissions through leaks during production and storage (Sherry et al., 2017). However, given a lack of detailed a priori point source information, we are limited to resolving emissions at a relatively coarse resolution as determined by the data with the NAME inversion method, or on a pre-determined grid with the FLEXPART inversion method. While our results indicate that Jiangsu, Shandong, and Shanghai are the areas which consistently show the largest CCl_4_ source per unit area, multiple industrial sources may be present in these areas, meaning we cannot prescribe a specific source or mechanism of emissions from location alone. In addition, the Gosan measurements show limited sensitivity to regions beyond the east coast of China (see supporting information Figure S1), and the posterior emissions distributions show significant uncertainty reductions mostly over eastern China and South Korea (see supporting information Figure S6). Thus, while we are able to robustly identify source regions in eastern China, there may be additional sources of CCl_4_ further west and south that cannot be identified through the Gosan measurements.

The resolved magnitudes and posterior spatial distribution of emissions indicate that there was a new source of emissions during the 8-year inversion interval. The 2009–2010 average (Figure 3a) shows regions of large emissions concentrated in a single region in the east of China around Shanghai and Jiangsu province. In addition to this region, large emissions are found further north in Shandong province in the 2013–2014 and 2015–2016 average estimates (Figures 3c–3d). This shift in emissions distribution is also identified in the FLEXPART estimates and is robust to a number of different assumptions about the prior distribution of emissions (see supporting information Text S3 and Figures S8–S10). There was not any significant change between 2009 and 2016 in the extent to which the Gosan data were sensitive to emissions from these regions (see supporting information Figure S1).

**Figure 4.**
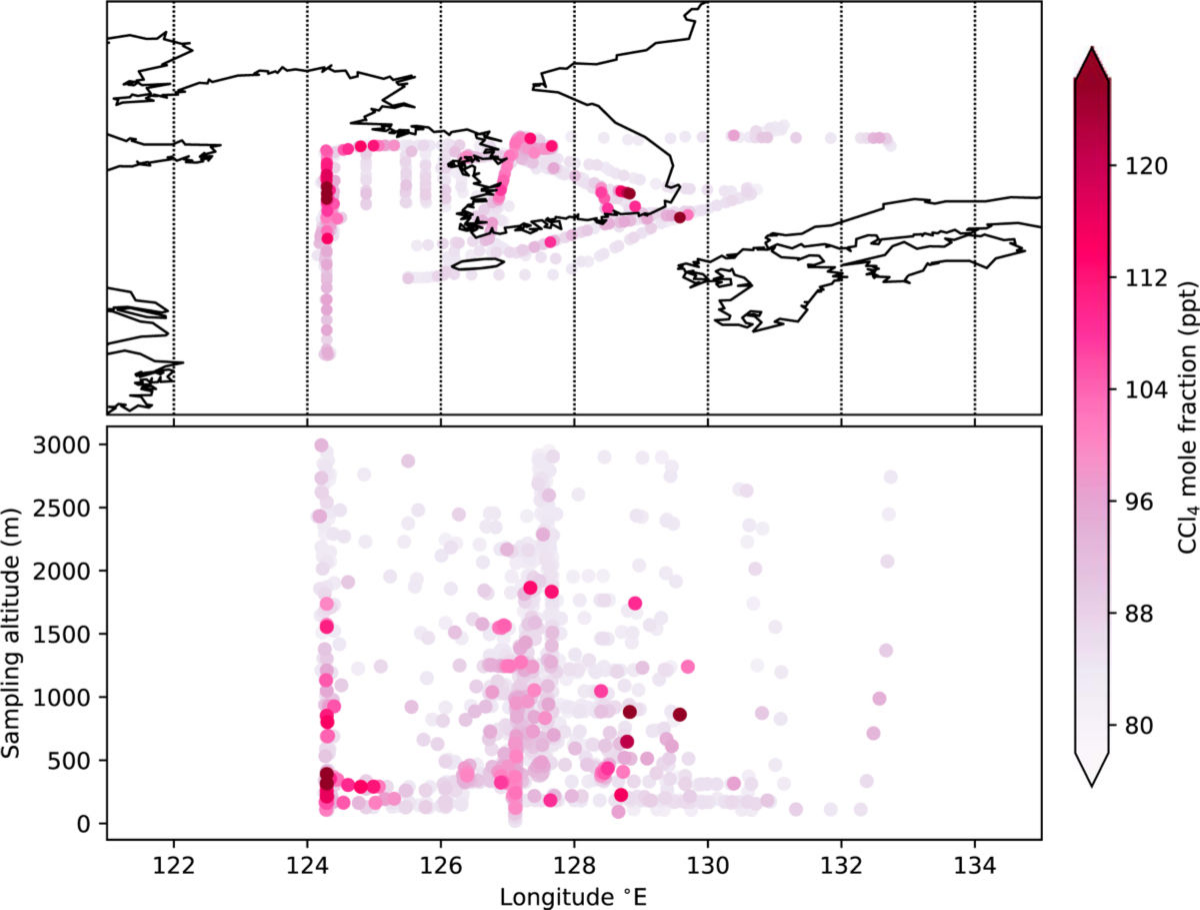
CCl_4_ mole fractions measured during the Korea-U.S. Air Quality aircraft campaign shown on a map projection in the top panel, and as a function of longitude and sample altitude in the bottom panel. The largest mole fractions were recorded below 500 m over the Yellow Sea and between 500 and 1,000 m over the southeast of the Korean Peninsula.

Our finding that emissions in the eastern Asian region surrounding Gosan are largely from China is supported by CCl_4_ measurements taken aboard the Korea-U.S. Air Quality (KORUS-AQ) flight campaign in May–June 2016. The KORUS-AQ data show some of the largest and most frequent mole fraction enhancements over the Yellow Sea between China and the Korean Peninsula (Figure 4). Back trajectory analysis using NAME shows that the largest mole fractions above 100 ppt (25% greater than background levels) were due to air received from the areas of high emissions identified in our inversions between Shanghai and the Shandong peninsula (see supporting information Figure S4).

We used the KORUS-AQ CCl_4_ data, in conjunction with the NAME model and inversion approach, to infer emissions between May and June 2016. Emissions were estimated using 898 data points representing the 1-min flask samples, averaged into 10-min bins. Although the results from this inversion are representative only of May–June 2016, the posterior emissions estimate from China is consistent with our Gosan-derived inversion results, identifying the same emissions areas in Jiangsu and Shandong provinces in China (see supporting information Figure S5). The posterior mean emissions from China were 16 (13–21) Gg/year, consistent with our Gosan-derived estimate using NAME for 2016 of 15 (11–20) Gg/year.

We find emissions from South Korea derived from the Gosan data to be small compared to China, averaging 0.4 (0.1–0.7) and 0.3 (0.1–0.5) Gg/year from NAME and FLEXPART, respectively, over the 8-year inversion period. This average is larger than the prior estimate of emissions of 0.2 Gg/year, although not statistically different. These relatively small emissions were concentrated in the southeast of the country (Figure 3). The KORUS-AQ samples identified one particularly large CCl_4_ peak of 138 ppt (175% of baseline value) in this region, in addition to another 10 measurements above 100 ppt that were at least 25% greater than baseline (Figure 4). Back trajectory analysis of these enhancements measured on the aircraft over the southeast of the Korean Peninsula show the air arrived from both China and South Korea, and inversion modeling using the KORUS-AQ data identified a region of emissions in the southeast of South Korea (see supporting information Figure S5). While this region of enhanced emissions in the southeast of South Korea was seen in the inversions using Gosan data, the feature is much more prominent in the KORUS-AQ data, likely as a result of a greater sensitivity to this area. The posterior mean emissions rate for South Korea from the KORUS-AQ inversion were 1.0 (0.8–1.2) Gg/year for May–June 2016, and together with the large observed KORUS-AQ mole fraction enhancements indicate that there may be a more significant source of emissions in the southeast of South Korea than can be seen from the Gosan data.

With the exception of 2011, we find emissions from Japan to be relatively small, with an 8-year mean of 0.6 (0.2–1.3) and 0.3 (0.0–1.0) Gg/year from NAME and FLEXPART, respectively, consistent with the bottom-up industrial estimate of emissions used as the prior (Sherry et al., 2017). In 2011, we find emissions from Japan to have been anomalously high, with the NAME-derived emissions in the 3-month summer period (June–August) of 6 (2–10) Gg/year, 10 times larger than the average emissions rate, with an overall annual emissions rate of 2.5 (1.1–4.1) Gg/year for 2011. The FLEXPART estimate of annual emissions from Japan for 2011 was also above average at 1.0 (0.4–1.6) Gg/year. The smaller annual emission rate indicates that the period of high emissions was relatively short-lived. Our inversion estimates identified a large signal centered on Honshu Island, north of Tokyo, shown in Figure 3b, consistent with the location of the Tohoku earthquake of March 2011. Although the emissions derived during the period of the earthquake itself remained small in March 2011, the enhancement observed 4 months later may be due to landfill disturbance during the cleanup operation. A similar summer 2011 maximum in emissions and mole fractions at three measurement sites in Japan has previously been noted for CFC-11, with the treatment of post earthquake debris being a likely cause (Saito et al., 2015). Notwithstanding this summer 2011 emissions event, our results indicate that the vast majority of emissions in the eastern Asia region came from China during this 8-year period.

## 4. Implications of Eastern Asia Emission Estimates

Based on the output of both NAME and FLEXPART inversions, our results show that eastern Asian emissions averaged 16 (9–24) Gg/year over the period 2009–2016, 24–58% of our estimated mean global emissions. Coupled to the regional estimates for the United States, Western Europe, and Australia from previous studies (Fraser et al., 2014; Graziosi et al., 2016; Hu et al., 2016), this combination of mean regional estimates sums up to 23 (13–34) Gg/year. These regional estimates overlap at the outer bounds of uncertainties with the global estimate of 38 (24–53) Gg/year averaged over the same period, helping to explain a large part of the discrepancy between inventory estimates and the global burden inferred from atmospheric measurements. However, the Gosan measurements are sensitive mostly to emissions from the east of China (supporting information Figures S1 and S6), which may mean there are additional sources present in China that cannot be identified from Gosan. In addition, there remain regions of the world, such as India, eastern Europe, Russia, and South America that are not well monitored, which could further add to this sum of regional emissions.

Whilst the outputs from the two different models and inversion methods are in broad agreement, there are some discrepancies between the NAME and FLEXPART results, with the NAME estimates notably higher in 2013 and 2015. Outputs from sensitivity tests on the impact of the prior emissions distribution, and inversion window used (shown in supporting information Text S3), indicate that these components of the inversion do not explain the difference between the two estimates. It is likely that the differences in emission estimates between NAME and FLEXPART are instead due to differences in transport between the models or in the estimation of baseline mole fraction contributions. Nevertheless, the use of one model over the other does not significantly affect our conclusions about the presence of ongoing CCl_4_ emissions from China that are larger than those estimated from bottom-up industrial estimates (Bie et al., 2017; Sherry et al., 2017).

The results of this work contrast with two separate studies using atmospheric data based on ISC to HCFC-22 (Bie et al., 2017; Park et al., 2018). The ISC results from Bie et al. (2017) used measurements of both compounds from Peking University in Beijing to derive emissions of around 4 Gg/year between 2011 and 2014, well below the lower uncertainty bounds of the estimates in this work. In contrast, the work of Park et al. (2018) used the same data used in this study from Gosan, along with estimates of HCFC-22 emissions to derive average emissions of CCl_4_ from China of 23.6 ± 7.1 Gg/year between 2011 and 2015, a total that is at the upper end of the estimates in this study. Potential biases in the ISC method lie in the emissions magnitude of the cotracer (HCFC-22, which was itself derived in an inversion), and in the assumption that emissions of CCl_4_ and HCFC-22 are colocated. Approximately 50 Gg/year HCFC-22 emissions have been estimated to be from the room air-conditioning sector in China in 2014 (Wang et al., 2016), accounting for around 40% of China’s total HCFC-22 emissions. Emissions from this sector should follow residential and business usage, and so may be broadly population based. In contrast, emissions of CCl_4_ are thought to be primarily from industrial sources (Park et al., 2018), and as we show in this work the spatial distribution of emissions is very different from a population distribution. Therefore, the assumption that sources of the two gases are colocated may not be valid and may help to explain the contrasting results between the different studies. Inversion modeling studies themselves may be prone to systematic errors in the transport model used, or the magnitude of the prior estimate of emissions (see supporting information Text S3 and Figure S8).

The discovery of a new source of emissions in Shandong province may potentially have wider implications linked to the recent discovery of a new source of CFC-11 into the atmosphere (Montzka et al., 2018), given that CCl_4_ is used in the production of CFC-11, and the timing of the change in source distribution after 2012 is consistent with an increased CFC-11 source around this time found by Montzka et al. (2018). More detailed work is required to confirm whether the findings of these two studies are linked. The posterior emissions derived here concur with the finding of Park et al. (2018) that there has not been a significant sustained reduction in emissions of CCl_4_ from China since the 2010 ban on production for dispersive uses came into force. Renewed efforts to control and remove emission sources of CCl_4_ around the world could help to enhance the decline of total tropospheric chlorine and speed up the subsequent recovery of ozone in the stratosphere.

## Supplementary Material

Supplementary Material

## Key Points:

- Emissions from eastern Asia region account for around 40% of global CCl_4_ emissions
- There has been no sustained decrease in emissions from the region since the introduction of production controls in 2010
- Main source regions are in Jiangsu and Shandong provinces of China
