# Supplementary material for: Continued Emissions of the Ozone-Depleting Substance Carbon Tetrachloride From Eastern Asia
Source: Geophys Res Lett. Author manuscript; Available in PMC 2020 Sep 30. (PMC7526663; doi:10.1029/2018gl079500)
Supplement: Supplementary Material [file NIHMS1517285-supplement-Supplementary_Material.pdf]

Supporting Information for "Continued Emissions of the Ozone Depleting Substance Carbon Tetrachloride from Eastern Asia"

M. F. Lunt[1], S. Park[2,3], S. Li[2], S. Henne[4], A. J. Manning[5], A. L. Ganesan[6], I. J. Simpson[7], D. R. Blake[7], Q. Liang[8], S. O'Doherty[1], C. M. Harth[9], J. Mühle[9], P. K. Salameh[9], R. F. Weiss[9], P. B. Krummel[10], P. J. Fraser[10], R. G. Prinn[11], S. Reimann[4], M. Rigby[1]

Corresponding author: M. F. Lunt, School of Chemistry, University of Bristol, Bristol BS8 1TS, UK. (mark.lunt@bristol.ac.uk)

[1]School of Chemistry, University of Bristol, Bristol BS8 1TS, UK

[2]Kyungpook Institute of Oceanography, College of Natural Sciences, Kyungpook National University, Daegu 41566, South Korea

[3]Department of Oceanography, School of Earth System Sciences, Kyungpook National University, Daegu 41566, South Korea

Contents of this file

1. Text S1 to S3

[4]Empa, Swiss Federal Laboratories for Materials Science and Technology, Überlandstrasse 129, Dübendorf, Switzerland

[5]Hadley Centre, UK Met Office, Exeter EX1 3PB, UK

[6]School of Geographical Sciences, University of Bristol, Bristol BS8 1SS, UK

[7]Department of Chemistry, University of California at Irvine (UC Irvine), Irvine, California 92697, USA

[8]Atmospheric Chemistry and Dynamics, NASA Goddard Space Flight Center, Greenbelt, Maryland 20771, USA

[9]Scripps Institution of Oceanography, University of California San Diego, La Jolla, California 92093, USA

[10]Climate Science Centre, CSIRO Oceans and Atmosphere, Aspendale, Victoria 3195, Australia

2. Figures S1 to S10

3. Tables S1 to S2

Introduction

The supporting information contains: (1) further information on the atmospheric data, models and inversion methodologies used in this study; (2) further information about the prior emissions totals and distributions used in this study, and outputs from sensitivity studies on the affect of these prior distributions; (3) results from sensitivity studies to different inversion set ups.

Text S1.

Measurements

High-frequency measurements of CCl_4 were performed at the Gosan station on Jeju Island, South Korea, which is part of the global AGAGE network [*Prinn et al.*, 2000]. The station is located approximately 300 km south-west of Busan, South Korea, 500 km north-east of Shanghai, China, and 300 km west of Nagasaki, Japan. Measurements of CCl_4 have been made using the Medusa Gas Chromatography Mass Spectrometry (GCMS) system [*Miller et al.*, 2008; *Arnold et al.*, 2012] since the latter half of 2008 until the present day. Measurements were made approximately every 2 hours with calibrations

[11]Centre for Global Change Science,
Massachusetts Institute of Technology,
Cambridge, Massachusetts 02139, USA

performed in between each air sample, calibrated on the SIO-05 scale. Air measured at Gosan predominantly arrives from the north-west or north-east directions carrying signals of pollution from China, South Korea and Japan [*Li et al.*, 2011, and Figure S1]. Plots of the 2-hourly Gosan data used in this work are shown in Figures S2 and S3.

Measurements of CCl_4 in the KORUS-AQ campaign were made on board the NASA DC-8 aircraft using a whole air sampling system [*Colman et al.*, 2001]. Flight paths covered tracks east, south and west of South Korea. In each of the 20 flights, 2L evacuated stainless-steel canisters were filled on a regular basis with a sampling period of approximately 1 minute every 4–5 minutes. Canisters were transported back to the UC Irvine (UCI) laboratory for analysis using the analytical procedure outlined in Simpson et al. [*Simpson et al.*, 2011]. The precision of the UCI CCl_4 measurements is 1% with an accuracy of 5%. Both AGAGE and UCI participated in the International Halocarbons in Air Comparison Experiment (IHALACE) [*Hall et al.*, 2014], showing that AGAGE and UCI CCl_4 mixing ratios agreed to within 2%, and scale factors of 0.973 (AGAGE) and 0.995 (UCI) were derived relative to CCl_4 measured by the National Oceanic and Atmospheric Administration (NOAA). Additional plots of the mean footprints of the most enhanced mole fractions in the KORUS-AQ data and the output of the inversion using the KORUS-AQ data are shown in Figures S4 and S5.

Text S2.

Atmospheric Models and Inversion Methods

NAME

In order to calculate the sensitivity relationship between emissions and simulated mole fractions, we used two different Lagrangian Particle Dispersion Models (LPDMs). The first of these, the Numerical Atmospheric dispersion Modelling Environment (NAME) [*Jones et al.*, 2007; *Manning et al.*, 2011], was driven by offline meteorological fields from the UK Met Office's global Unified Model (UM) [*Cullen*, 1993], providing meteorological fields at approximately 25 km horizontal resolution prior to 2014 and at 17 km thereafter, on a 3-hourly timescale. Model particles were released from the location of the measurement site, at a height of 10 ±10 m a.g.l. at a rate of 20,000 particles per hour. A new release was instigated every 2 hours to match the approximate temporal resolution of the data. Particles were tracked backwards in time for 30 days, or until they left the computational domain, whichever occurred sooner. The NAME computational domain spanned from 55°E–168 °W and 5 °S–74 °N, and the output fields were at 0.234° x 0.352° resolution. The model output gave the integrated residence time of model particles from each horizontal grid cell in the surface layer, which was defined as 0–40 m, providing a sensitivity footprint of the mole fraction at the measurement site to emissions from any of the model output grid cells for each 2 hour observation period. These footprints, **H** were then related to the mole fraction data, **y** following:

$$\mathbf{y} = \mathbf{H} \cdot \mathbf{x}, \tag{1}$$

where $\mathbf{x}$ is a parameters vector incorporating emissions. The available 2-hourly observations from Gosan were filtered based on a combination of the contribution to the total footprint of the 25 grid cells surrounding the release point, and the modelled boundary layer height. Times when the air arriving at the site had a local contribution of greater than 10% of the total, or when the boundary layer was less than 200 m were not included in the analysis. NAME was also used to produce back-trajectories of the mole fractions measured during the KORUS-AQ campaign. Model particle releases were centred on the 3D location of the aircraft at each given flask sampling measurement point. Particles were released at a rate of 4,000 particles per minute for a period of 1 minute and tracked backwards in time for 30 model days.

The NAME footprints accounted only for the sensitivity to emissions within the NAME computational domain. To calculate the sensitivity to background atmospheric mole fractions (which orginated from outside the NAME domain) we used model estimates of the mole fraction field at the boundaries of the computational domain simulated using the NASA 3-D GEOS Chemistry Climate Model [*Liang et al.*, 2014]. The global model mole fraction field in each grid box at the edge of the NAME domain was multiplied by the fraction of NAME particles that exited the domain from the respective grid box, to create a time series of background concentrations at the measurement site. Whilst the global model estimates provided a realistic monthly climatological distribution of CCl_4 at the boundaries of the domain, the absolute magnitude of the global field was allowed to vary in our inversion system. For each month of the inversion a parameter was optimized that described a scaling factor applied to this global mole fraction field.

NAME Inversion Method

To estimate CCl_4 emissions using NAME we used a transdimensional hierarchical Bayesian inversion approach described in detail in *Lunt et al.* [2016]. A prior estimate of emissions, $\mathbf{x}$ was informed by the data, $\mathbf{y}$, and the estimate of emissions was subsequently updated. In addition, we also estimated the number of unknown parameters for which to solve, k, and additional hyper-parameters, θ, following the transdimensional hierarchical Bayesian approach outlined in *Lunt et al.* [2016] and given by Eq. 2:

$$\rho(\mathbf{x}, \theta_{\mathbf{x}}, \theta_{\mathbf{y}}, k \mid \mathbf{y}) \propto \rho(\mathbf{y} \mid \mathbf{x}, \theta_{\mathbf{y}}, k) \cdot \rho(\mathbf{x} \mid \theta_{\mathbf{x}}, k) \cdot \rho(\theta_{\mathbf{x}}) \cdot \rho(\theta_{\mathbf{y}}) \cdot \rho(k). \quad (2)$$

Where $\theta_{\mathbf{x}}$ represents the hyper-parameters describing the prior emissions uncertainty covariance matrix, and $\theta_{\mathbf{y}}$ the model-measurement uncertainty covariance matrix.

This transdimensional, hierarchical approach takes account of the fact that the posterior probability of emissions, given on the left hand side of equation 2 is dependent upon the number of unknowns and the structure of the uncertainty covariance matrices [*Ganesan et al.*, 2014; *Lunt et al.*, 2016]. The posterior PDF was estimated using a reversible-jump Markov chain Monte Carlo (rj-MCMC) algorithm [*Green*, 1995; *Lunt et al.*, 2016]. This approach allows the number of unknowns (basis functions) to itself be an unknown which is informed by the data. The approach has the advantage that changes to the prior estimate of emissions are made at a resolution that the data allow, more accurately reflecting the information content of the data. Unlike most Bayesian inversion approaches, we do not have to pre-define a grid on which emissions will be resolved, an approach that should

be well-suited to the case of CCl_4, where prior information on the location of sources is limited.

A uniform PDF was used to describe the number of unknowns, and allowed to vary between 5 and 150 regions. Hyper-parameters describing the prior emissions uncertainty, model uncertainty and model error correlation time scale were each allowed to vary and were solved for in the inversion. The prior uncertainty on each spatial basis function was set to 100% of the prior mean, but described by a uniform PDF which had minimum and maximum bounds of 50 and 400%, reflecting the large uncertainty in the prior estimate of emissions. The model-measurement uncertainty was described by a uniform PDF which had minimum and maximum bounds of 0.5 and 40 ppt respectively. Different values for model-measurement uncertainty at Gosan were estimated every 5 days, a time scale roughly equivalent to synoptic scale systems. The magnitude of the resolved posterior model-measurement uncertainties are shown by the shading in figures S2 and S3, and averaged 2.5 ppt across all inversions. The correlation length scale, describing the covariance between model-measurement errors was described by a uniform PDF with bounds of 0.1 and 96 hours. The optimized posterior mean over all inversions was 14 hours.

The first 50,000 iterations of the rj-MCMC algorithm were discarded as a burn-in period, with the chain run for a further 200,000 iterations, and thinned to store every 100th iteration (2000 samples). Automatic tuning of the proposal perturbation sizes for the parameters and hyper-parameter updates was performed during the burn-in period to maintain acceptance rates of between 20–50%, ensuring efficient exploration of the target distribution [*Tarantola*, 2005]. Convergence of the parameters in reversible-jump MCMC

is more difficult to assess than conventional MCMC since, due to the changing basis function definitions, the value of a basis function at the first iteration does not necessarily represent the same quantity as the value at, for example, the one-hundredth iteration. In this work we examined the negative log-likelihood to determine that the chain had converged around a stationary part. Two separate samples, the first 10% and last 50% of each chain returned means that were within 1% of each other in each case.

Independent 3-monthly inversions were performed with emissions assumed constant over each 3-month period. The mean output of these inversions was used to form the annual means, with the annual uncertainties based on the assumption that posterior uncertainties between the independent inversions in each year were fully correlated. The 3-month inversion period was chosen to reduce the temporal aggregation of emissions compared to an annual inversion, and as a result of the computational constraints of performing annual inversions using 2-hourly data. An additional test was performed to assess the impact of performing the inversion using a 1-year inversion window, albeit at reduced data frequency of 24 rather than 2 hours. The average posterior China emissions from this inversion set-up were 20 (14–26) Gg yr^{-1}, suggesting that the temporal aggregation of the inversion does not have a significant impact on the results and does not explain why the posterior emissions from NAME are larger than those from FLEXPART (see Figure S8). Only posterior emissions from China east of 104°E were included in our analysis, an area that accounts for approximately 80% of the population of China [*CIESIN*, 2016]. Although emissions were assigned west of this boundary and optimized using the data, due to the distance from the measurement site of over 2000 km, and hence low sensitivity,

there was negligible change from the prior and hence they are not included in our analysis. Including the whole of China in the inversion led to a mean result that was 15% larger, although not statistically different.

FLEXPART

Surface source sensitivities for the site Gosan were derived with another LPDM, FLEXPART, driven by analysis/forecasts from the operational runs of the Integrated Forecast Systems of the European Centre for Medium-Range Weather Forecasts (ECMWF). The horizontal resolution of the input data was 0.2°x0.2° over the area of interest in eastern Asia and 1°x1° elsewhere. In 3-hourly intervals, 50,000 model particles were released at the location of the Gosan measurement site and traced back in time for 240 hours. Source sensitivities were obtained by calculating the residence times of the model particles within a regular geographic grid covering the Eurasian continent.

FLEXPART Inversion Method

The regional scale inversion method using FLEXPART also follows a Bayesian approach and is described in detail by *Henne et al.* [2016], where it was applied to methane emissions in Switzerland, and has also been applied with varying set-ups to estimate halocarbon emissions in Europe [*Brunner et al.*, 2017; *Schoenenberger et al.*, 2017]. The spatial grid did not exhibit uniform basis function sizes for which emissions were estimated, but was constructed following the average simulated source sensitivity, with smaller basis functions where source sensitivities were larger, and larger basis functions where source sensitivities were smaller. Like NAME, the FLEXPART-simulated mole fractions consisted of a directly simulated regional contribution and a baseline contribution. The baseline contri-

bution was estimated from a statistical fit to the observed time series at Gosan [*Ruckstuhl et al.*, 2012]. The resulting baseline was optimised at 5-day nodes as part of the state vector [*Stohl et al.*, 2009], using linear interpolation for times between the baseline nodes. The inversion was applied to yearly batches of observations, therefore yielding mean emissions for each year in the investigated period from 2009 to 2016. All valid observations from the Gosan observatory were used in the inversion, no additional filtering by time-of-day or wind direction was applied.

The structure and the values of the covariance matrices for the *a priori* emission uncertainties and model-measurement uncertainties were described by a set of parameters characterising absolute uncertainty levels and spatio-temporal correlations in the uncertainties [*Henne et al.*, 2016]. These parameters included the treatment of autocorrelation in the observations with a temporal correlation length of 0.2 days. This value was set based on an exponential fit to the empirical auto-correlation function of the *a priori* model residuals. Furthermore, the relative total uncertainty, σ_E, and spatial correlation length scale, L, of the prior emissions, the absolute uncertainty, σ_b, and the temporal correlation length scale, τ_b, of the prior baseline, and two parameters describing the absolute and relative (to simulated sensitivity) data-mismatch uncertainty, σ_{min}, and σ_{srr}, were obtained from a log-likelihood (LLH) maximum search [*Berchet et al.*, 2013; *Henne et al.*, 2016; *Michalak et al.*, 2005], which was carried out separately for each annual inversion batch. The resulting parameters varied slightly from year to year (see Table S1). The estimated relative uncertainty of the *a priori* emissions ranged from 56% in 2012 to 97% in 2010. With the exception of 2009, the correlation length scale of the *a priori* emis-

sion uncertainty ranged from 158 to 454 km. The relatively small value for the baseline uncertainty (smaller 1 ppt for all years except 2012) indicates a robust estimation of the prior baseline. In consequence, a-posteriori adjustments of the baseline were small and had only minor influence on the inversion results. Although not sufficient in itself, the χ^2 index [*Berchet et al.*, 2013] offers a useful indication if the covariance matrices are well balanced, in which case a value close to 1 should be obtained. The latter was the case for all years covered by the inversion.

Text S3

Prior Emissions

The *a priori* estimate of emissions for China, Japan and South Korea of 7.3, 0.6 and 0.2 Gg yr^{-1} respectively used previously reported country totals from *Sherry et al.* [2017] in both NAME and FLEXPART inversions. However, these country totals were distributed differently in these two different methods. For the NAME inversions CCl_4 emissions were distributed evenly throughout each country. *A priori* emissions from countries other than China, Japan and South Korea were set to small values of 0.05 Gg yr^{-1} but with significant uncertainties, enabling the data to update them as required.

The FLEXPART inversions used the same *a priori* country totals for the three main countries of interest, but spatially disaggregated these emissions proportionally to population densities [*CIESIN*, 2016]. For North Korea a first guess total emission of 0.1 Gg yr^{-1} was assigned. For all other countries in the transport domain an average per capita emission factor was ascribed based on the *a priori* values for China, Japan and South Korea. *A priori* emissions were the same for each year. Since the inversion assumed

normally distributed uncertainties and solved the Bayesian cost function analytically, negative a-posteriori emissions were not explicitly excluded from the solution space. To assure positive a-posteriori emissions the approach iteratively adjusted *a priori* uncertainties for grid cells with negative a-posteriori emissions [*Stohl et al.*, 2009].

The impact of different assumptions about this *a priori* distribution of emissions, and the degree to which it might lead to differences between the NAME and FLEXPART estimates were explored, by considering a number of different *a priori* emissions in the NAME inversions, shown in Figure S7, and outlined below:

1. Population-weighted prior. Forcing emissions used in the NAME inversions to be distributed according to population density (the set-up most similar to FLEXPART inversions) resulted in a mean emission from China of 16 (10–23) Gg yr^{-1}, indicating that the discrepancy between the two different inversion approaches was not due to the definition of the *a priori* emissions distribution.

2. Night lights filtered prior. We used a night-lights filter based on stable night-light information from NASA's Defense Meteorological Satellite Program's Operational Linescan System (DMSP-OLS) sensors (available at https://ngdc.noaa.gov/eog/dmsp/dmsp.html). This filter limited emissions to reside in grid boxes where the intensity of light was greater than zero, where a value of zero represents areas of no light, thereby attempting to limit the plausible distribution of emissions to areas of economic activity. The night lights filter was used in a purely binary sense, with a grid cell either containing emissions or not, and *a priori* emissions were not distributed according to the intensity of light. This is in contrast to the population-weighted prior where emissions were distributed by the popula-

tion density in each grid cell. Restricting emissions to reside in grid cells with night-lights resulted in mean emissions that were statistically similar to the case of no restriction of 17 (11–25) Gg yr^{-1} (see Figure S8). Furthermore, the same change in spatial distribution of emissions between 2009-2010 and 2015–2016 was also found (Figures S9 and S10).

3. Four times larger prior. A further test explored the dependence of the posterior emission totals for China on the magnitude of the prior emissions by increasing this prior magnitude by a multiple of 4, to 29.2 Gg yr^{-1}. The posterior mean for China between 2009–2016 was found to be 24 (16–36) Gg yr^{-1} in this case. This increase of the prior emissions total resulted in the largest change in posterior emissions of any inversion set-up, likely reflecting the fact that large areas of the spatial inversion domain were not well constrained by the data, and thus there was little change from the *a priori* definition. The results of this test show that there is some dependence of our inversion estimates on the prior emissions total used. Nevertheless, both prior totals used in our NAME inversions for China of 7.3 Gg yr^{-1} and 29.2 Gg yr^{-1} returned posterior means that were in the range 16–25 Gg yr^{-1}, with overlapping uncertainty bounds.

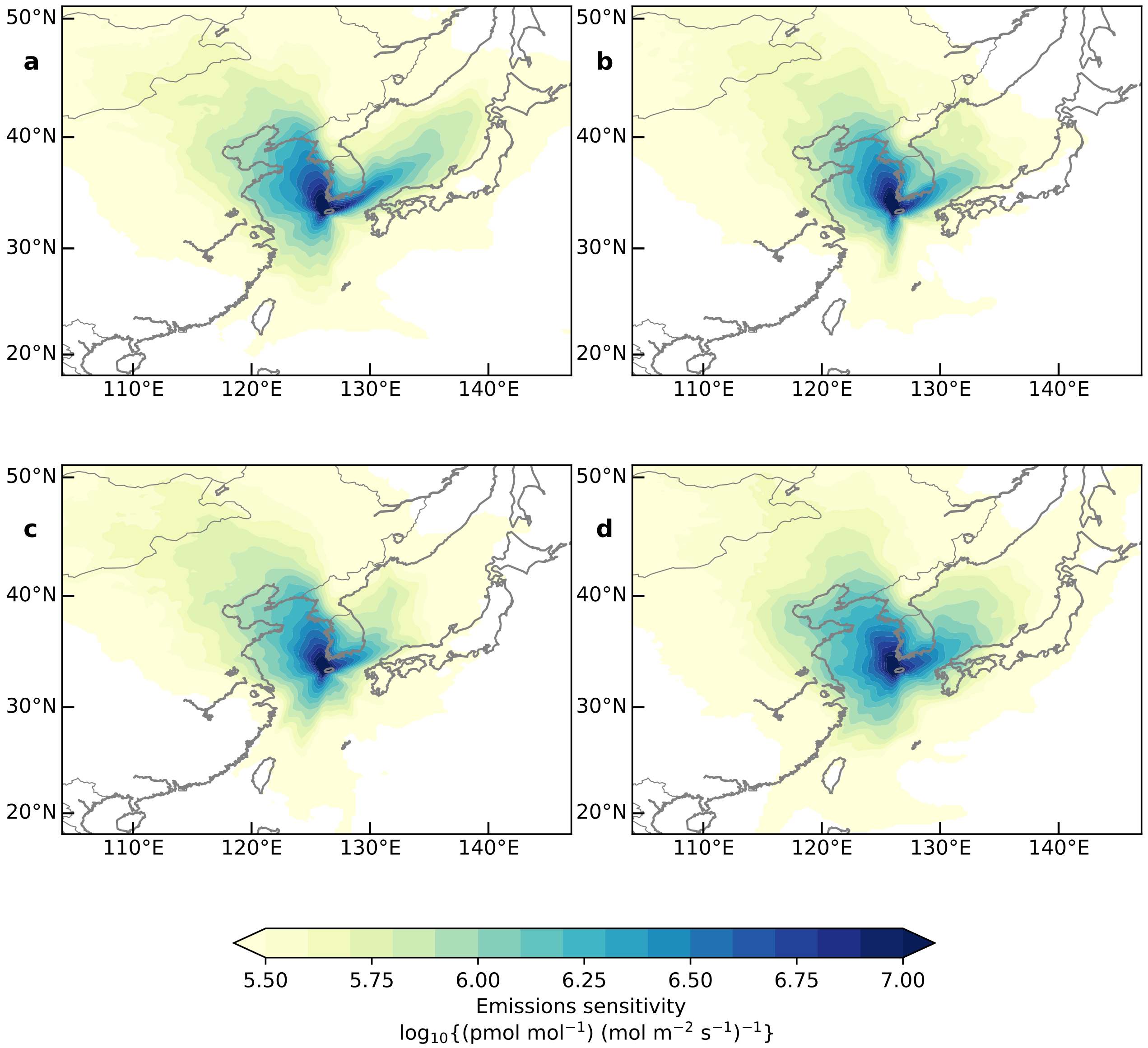

Figure S1. Plots of the mean NAME footprint at Gosan for **a** 2009–2010; **b** 2011–2012; **c** 2013–2014; **d** 2015–2016. The extent to which the data is sensitive to emissions areas is relatively invariant over time, although there is limited sensitivity to areas beyond South Korea and the east coast of China.

Table S1. FLEXPART inversion covariance and inversion parameters (for details see text). N_o: number of observations, N_g: number of grid cells in inversion grid, N_b: number of background nodes, χ^2: χ^2 index as defined in *Berchet et al.* [2013], d_f: degree of freedom estimated as the trace of the averaging kernel

Year	σ_{srr}	σ_{min}	σ_E	L	σ_b	τ_b	N_o	N_g	N_b	χ^2	d_f
2009	4.27	0.37	0.69	1	0.16	90	1660	355	53	1.07	17
2010	1.60	0.84	0.97	210	0.18	27	1891	421	54	1.05	34
2011	1.71	0.71	0.84	194	0.16	24	1593	358	54	1.04	30
2012	1.28	0.86	0.56	158	4.28	4	1838	418	54	0.98	73
2013	1.89	0.90	0.78	454	0.15	18	2118	430	54	1.04	22
2014	2.32	1.31	0.85	412	0.16	6	2223	496	55	1.04	18
2015	1.96	1.33	0.85	214	0.15	1	1544	364	52	1.06	23
2016	1.94	0.64	0.94	249	0.15	90	1256	340	40	1.05	22

Table S2. NAME and FLEXPART emission estimates for China, South Korea and Japan. All totals in Gg yr^{-1}.

Year	China (NAME)	China (FLEXPART)	S. Korea (NAME)	S. Korea (FLEXPART)	Japan (NAME)	Japan (FLEXPART)
2009	13 (9–18)	11 (3–19)	0.1 (0.0–0.4)	0.3 (0.0–0.6)	0.5 (0.1–1.3)	0.6 (0.0–1.5)
2010	13 (8–18)	11 (7–15)	0.4 (0.2–0.7)	0.6 (0.4–0.8)	0.7 (0.2–1.8)	0.2 (0.0–0.9)
2011	10 (6–18)	11 (5–16)	0.2 (0.1–0.4)	0.3 (0.2–0.5)	2.5 (1.1–4.1)	1.0 (0.4–1.6)
2012	12 (8–18)	12 (7–17)	0.2 (0.0–0.6)	0.4 (0.3–0.6)	0.5 (0.1–1.2)	0.2 (0.0–1.0)
2013	25 (17–35)	16 (10–22)	0.5 (0.2–0.7)	0.3 (0.1–0.4)	0.6 (0.2–1.6)	0.1 (0.0–0.7)
2014	18 (13–23)	15 (9–21)	0.4 (0.2–0.6)	0.2 (0.0–0.3)	0.6 (0.2–1.3)	0.1 (0.0–0.8)
2015	27 (17–37)	13 (7–19)	0.1 (0.0–0.3)	0.3 (0.1–0.5)	0.5 (0.1–1.2)	0.2 (0.0–0.9)
2016	15 (11–20)	13 (5–20)	0.3 (0.1–0.6)	0.3 (0.0–0.5)	0.5 (0.1–1.1)	0.3 (0.0–1.0)

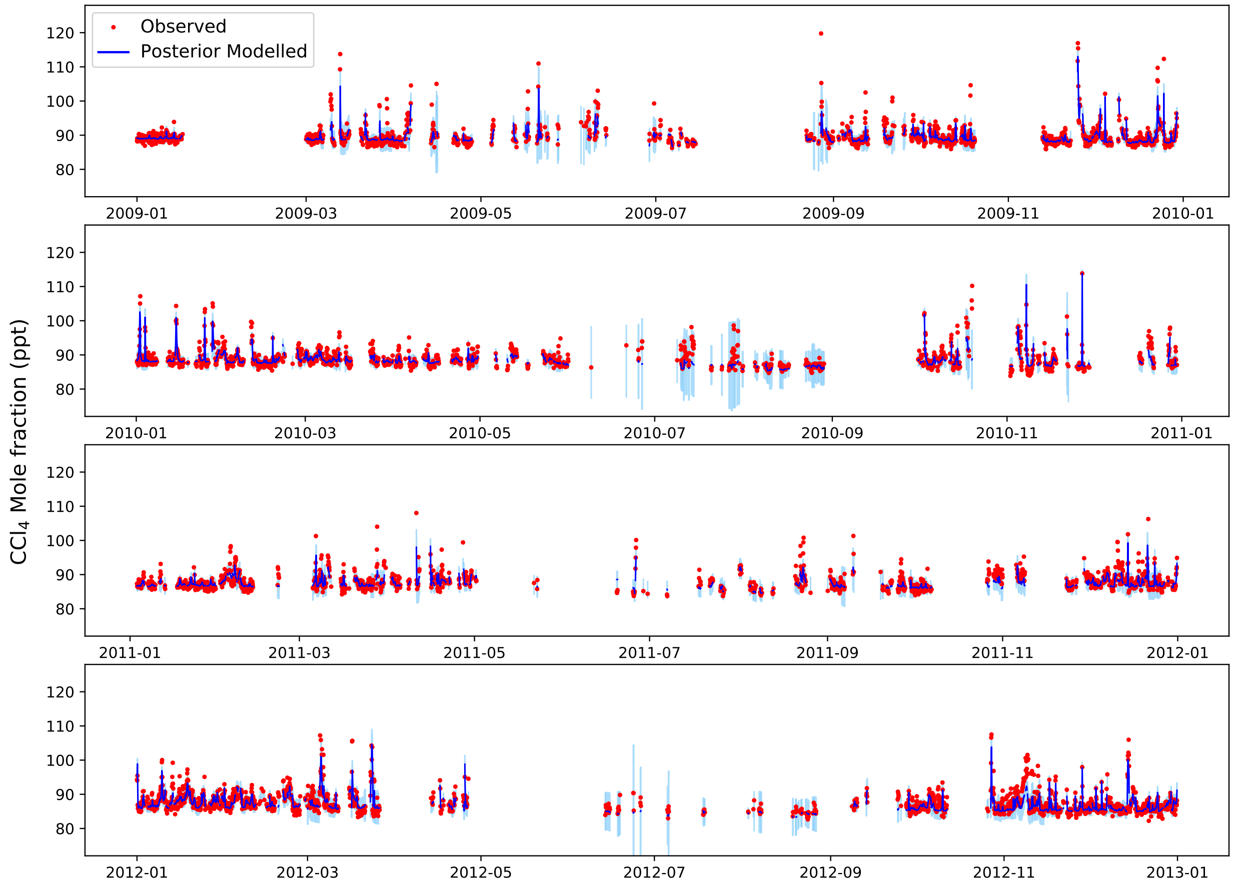

Figure S2. Time series of 2-hour averaged mole fraction observations and model output for Gosan, South Korea for 2009–2012 inclusive. Observed values are in red, and posterior mean modelled values in blue. The modelled output are from the NAME inversion results in the main text. The blue shading shows the 1σ standard deviation of the model-measurement uncertainty, and represents the relative weighting of each data point used in the inversions. Shaded values well below observed baseline mole fractions are visible due to the symmetric Gaussian nature of the uncertainty, and is most likely indicative of the magnitude of uncertainties in the above-baseline pollution events. Gaps in the data were caused by a combination of instrument downtime and the observation selection criteria applied in our NAME inversions.

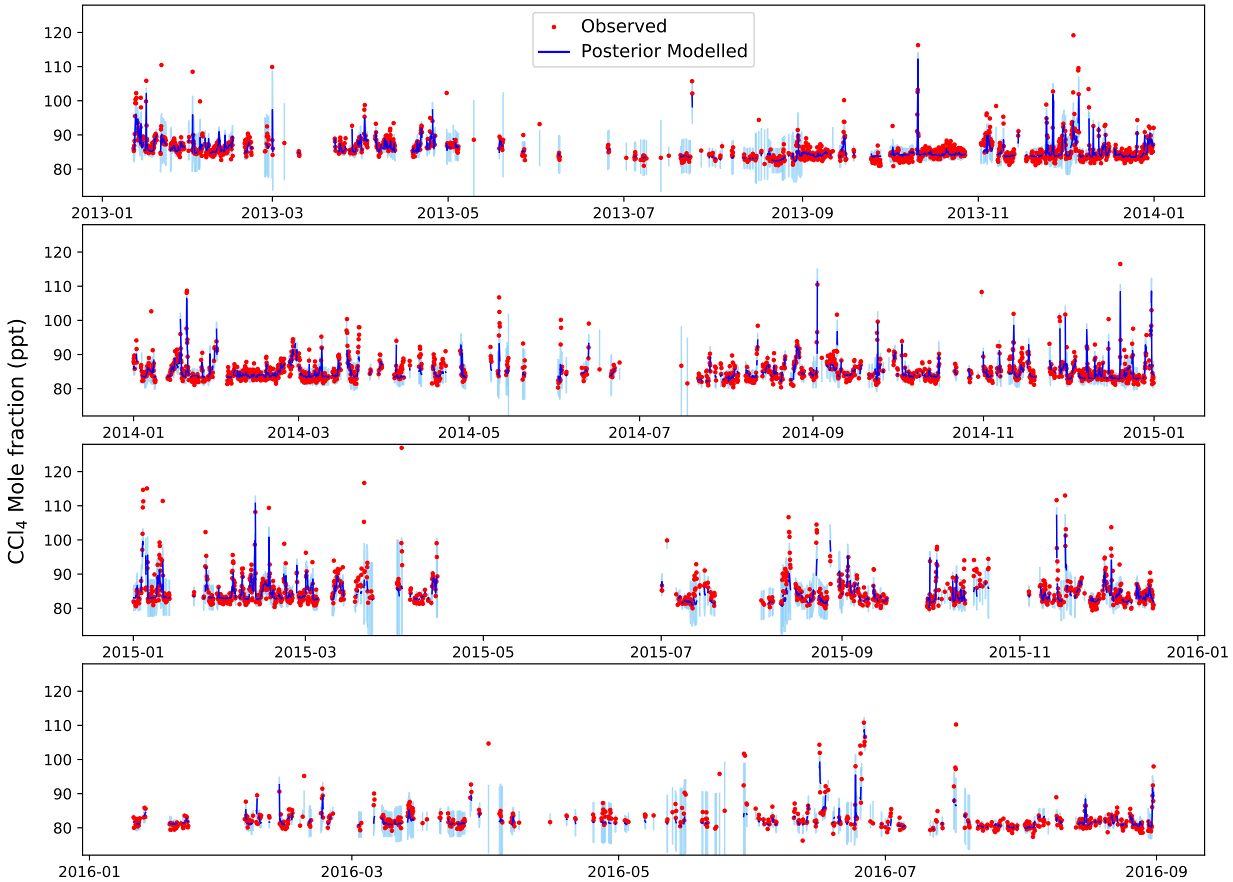

Figure S3. As Figure S2 for 2013–2016.

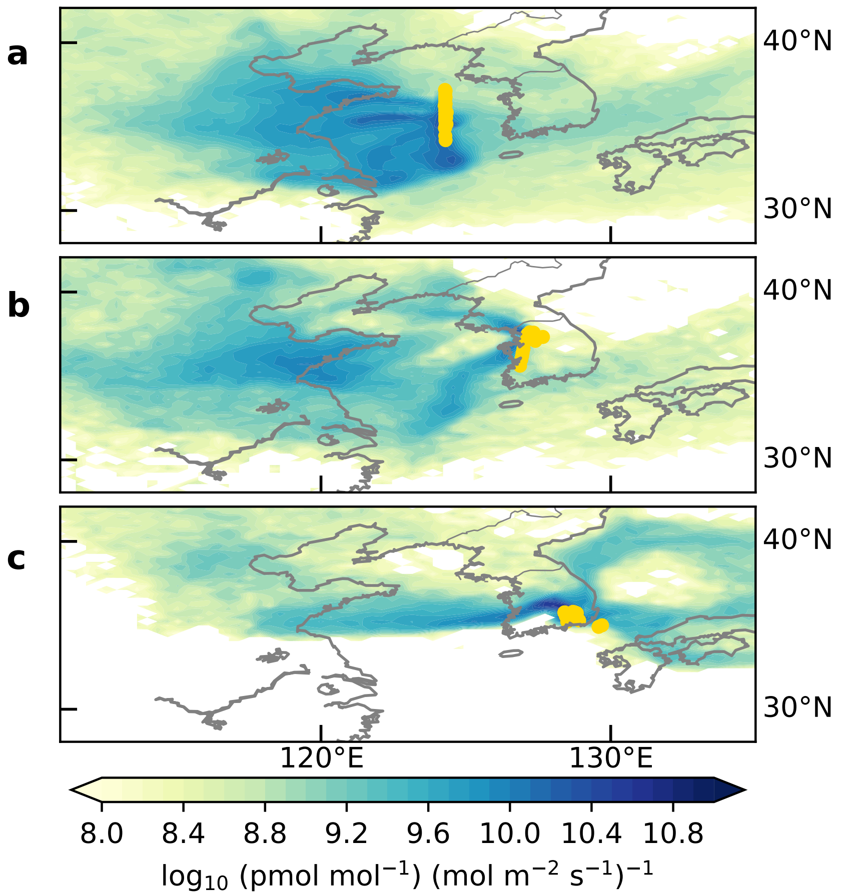

Figure S4. NAME back-trajectories for mole fractions of CCl_4 measured during the KORUS-AQ campaign that were greater than 100 ppt in three longitude bands: (**a**) measurements west of 125°E; (**b**) measurements between 126°E and 128°E and (**c**) east of 128°E. The back-trajectories show the air arrived from the regions of high emissions in China identified in our inversions using Gosan data. The more easterly points show enhancements may also be due to air arriving from over South Korea.

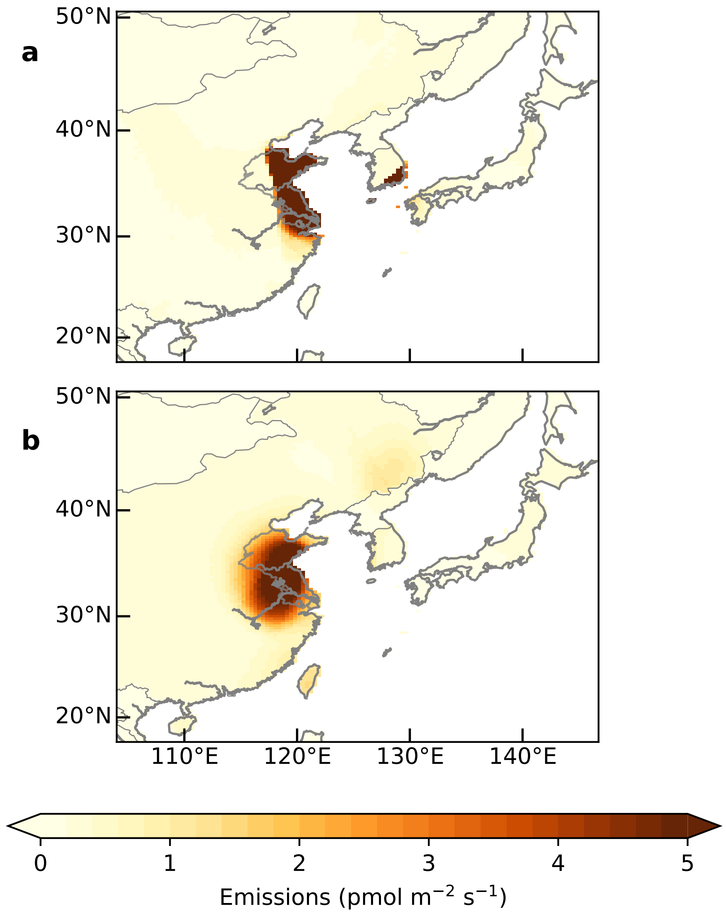

Figure S5. (**a**) Map of the posterior distribution of emissions derived using KORUS-AQ data and the NAME model and inversion approach for May–June 2016. The *a priori* emissions distribution was assumed flat. (**b**) The posterior distribution of emissions derived using Gosan data during March–August 2016.

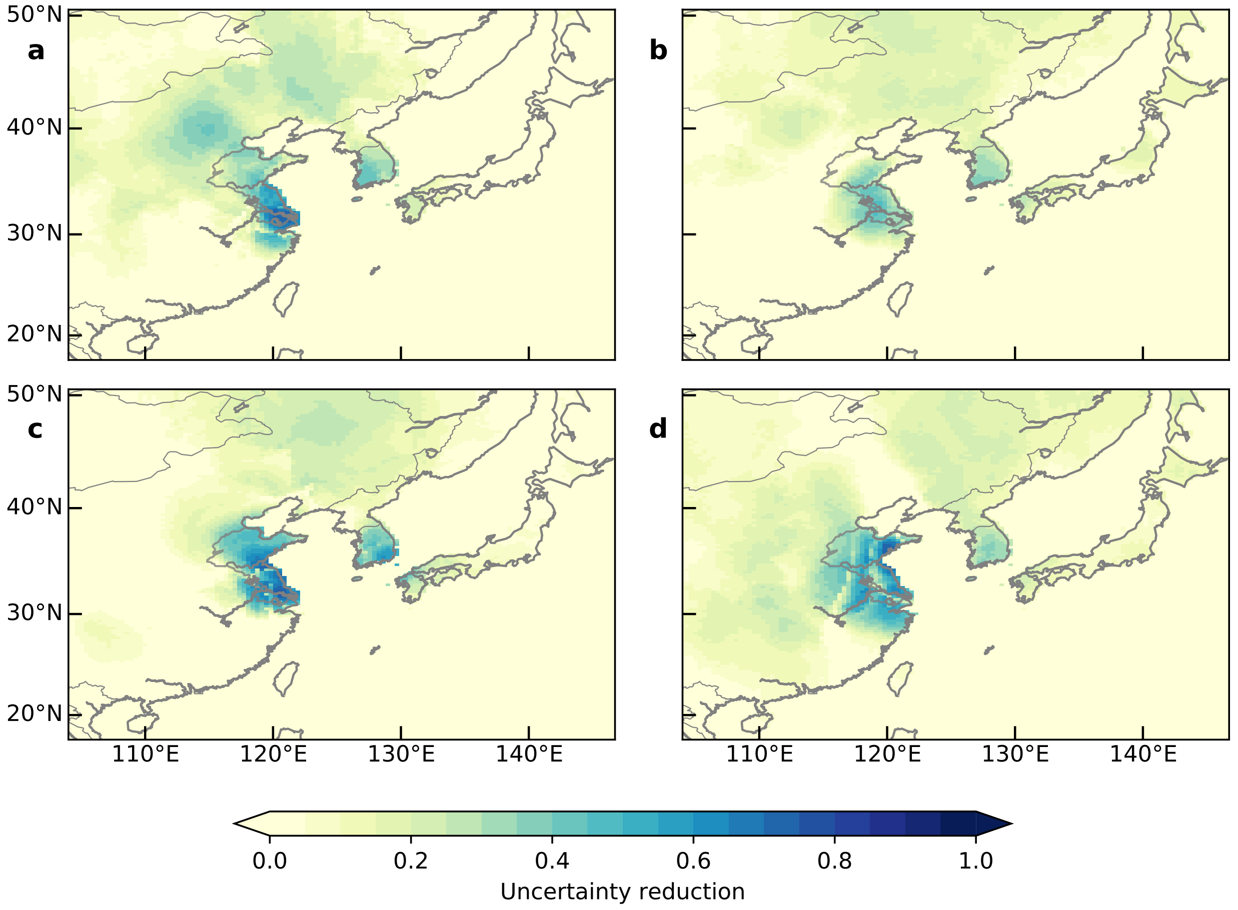

Figure S6. Maps of the average posterior uncertainty reduction for 2-year periods from the NAME inversions for: (a) 2009–2010; (b) 2011–2012; (c) 2013–2014 and (d) 2015–2016. The uncertainty reduction is given by: $1 - \frac{posterioruncertainty}{prioruncertainty}$, where a value of 1 (dark blue) indicates zero posterior uncertainty and a value of 0 (light yellow) represents no reduction from the prior uncertainty. Areas of uncertainty reduction are largely restricted to South Korea and the east of China.

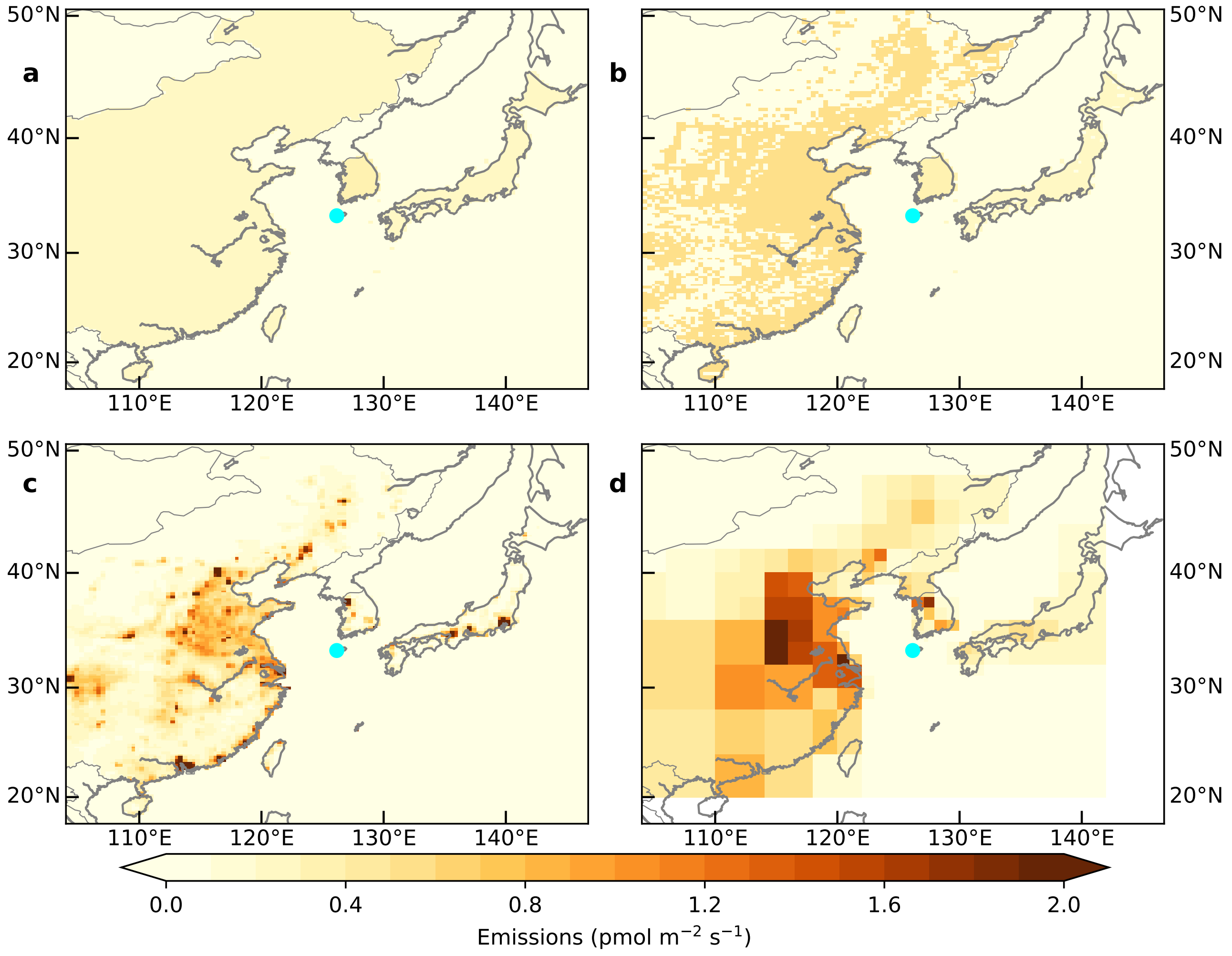

Figure S7. Maps of the *a priori* distribution of emissions for 4 different inversion set-ups, showing: (**a**) flat prior used in NAME inversions; (**b**) prior emissions limited to where night light intensity was greater than 0, used in NAME inversions; (**c**) population-weighted prior distribution used in NAME inversions (**d**) population-weighted prior distribution on a fixed grid used in FLEXPART inversions. Note the different scale to Figures S9 and S10. The Gosan measurement site is shown by the cyan dot.

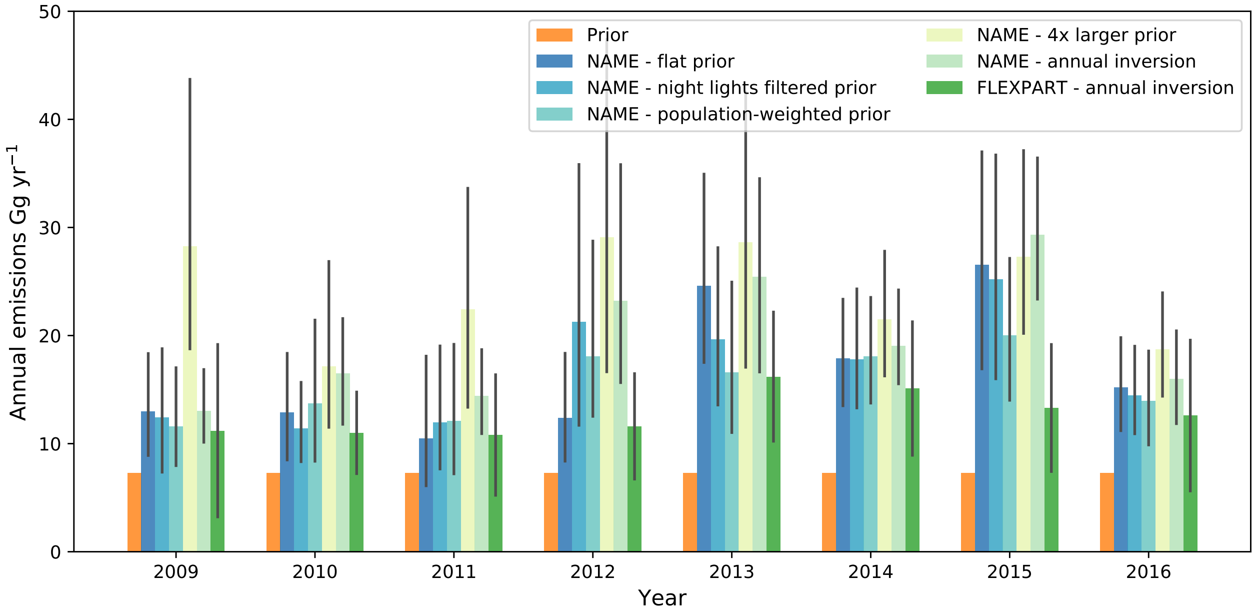

Figure S8. Bar plot of emissions from China between 2009–2016, under different *a priori* assumptions. Orange bars show the prior total used of 7.3 Gg yr^{-1}. The blue and yellow bars show the posterior annual means for different NAME inversions showing: (1) The reference case presented in the main text using a flat distribution; (2) Prior emissions limited to where night light intensity was greater than 0; (3) Prior emissions distributed using population weighting; (4) Prior emissions four times larger than the reference case; (5) Annual inversion window. FLEXPART inversions using a population-weighted prior distribution are in green. Uncertainties from NAME represent the 90% confidence interval, whilst FLEXPART uncertainties represent the 2σ uncertainty level.

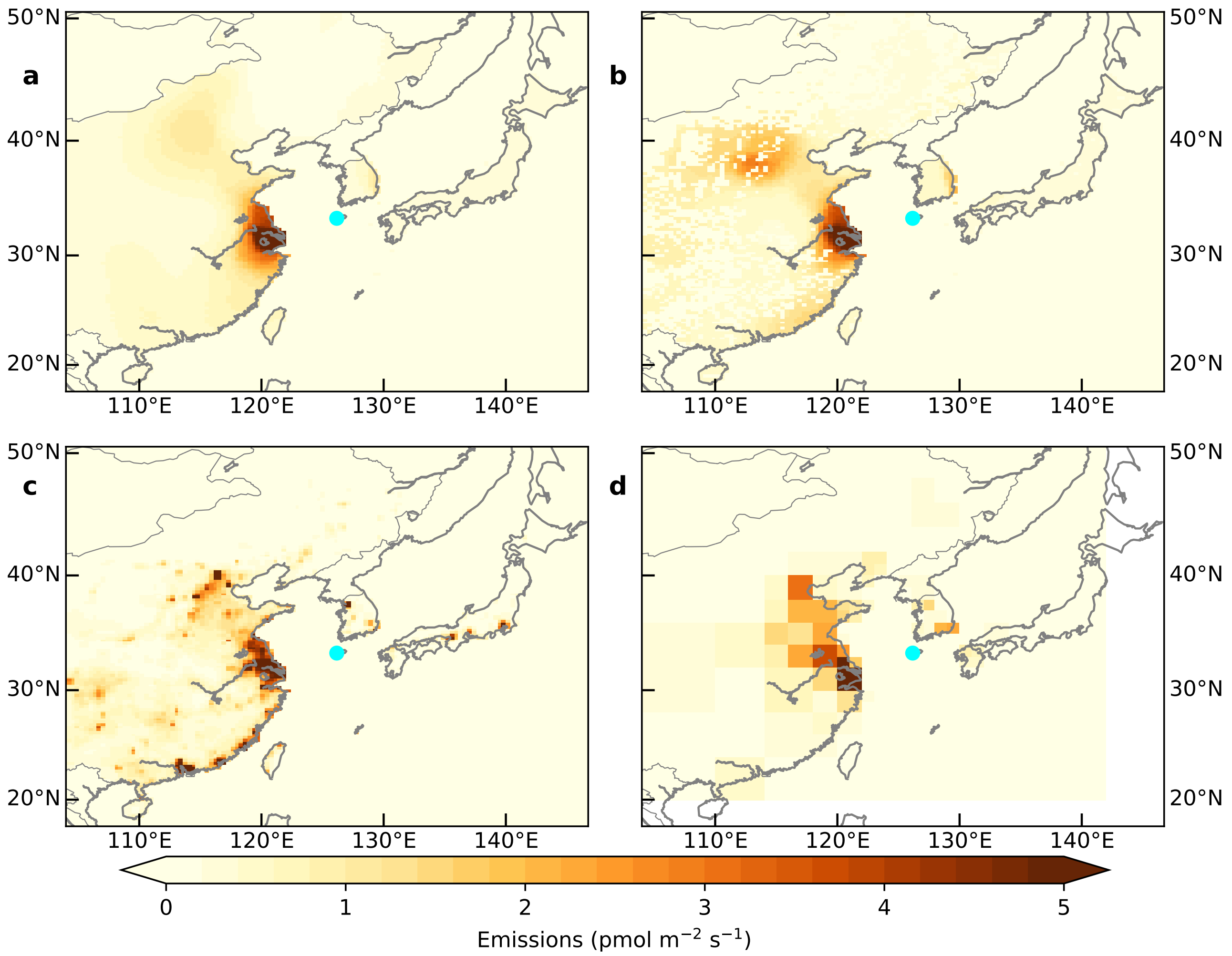

Figure S9. Maps of the posterior distribution of emissions averaged over 2009–2010, for 4 different inversion set-ups, showing: (**a**) NAME flat prior; (**b**) NAME with emissions limited to where night light intensity was greater than 0, (**c**) NAME with population-weighted prior distribution (**d**) FLEXPART with population-weighted prior distribution on a fixed grid. The Gosan measurement site is shown by the cyan dot.

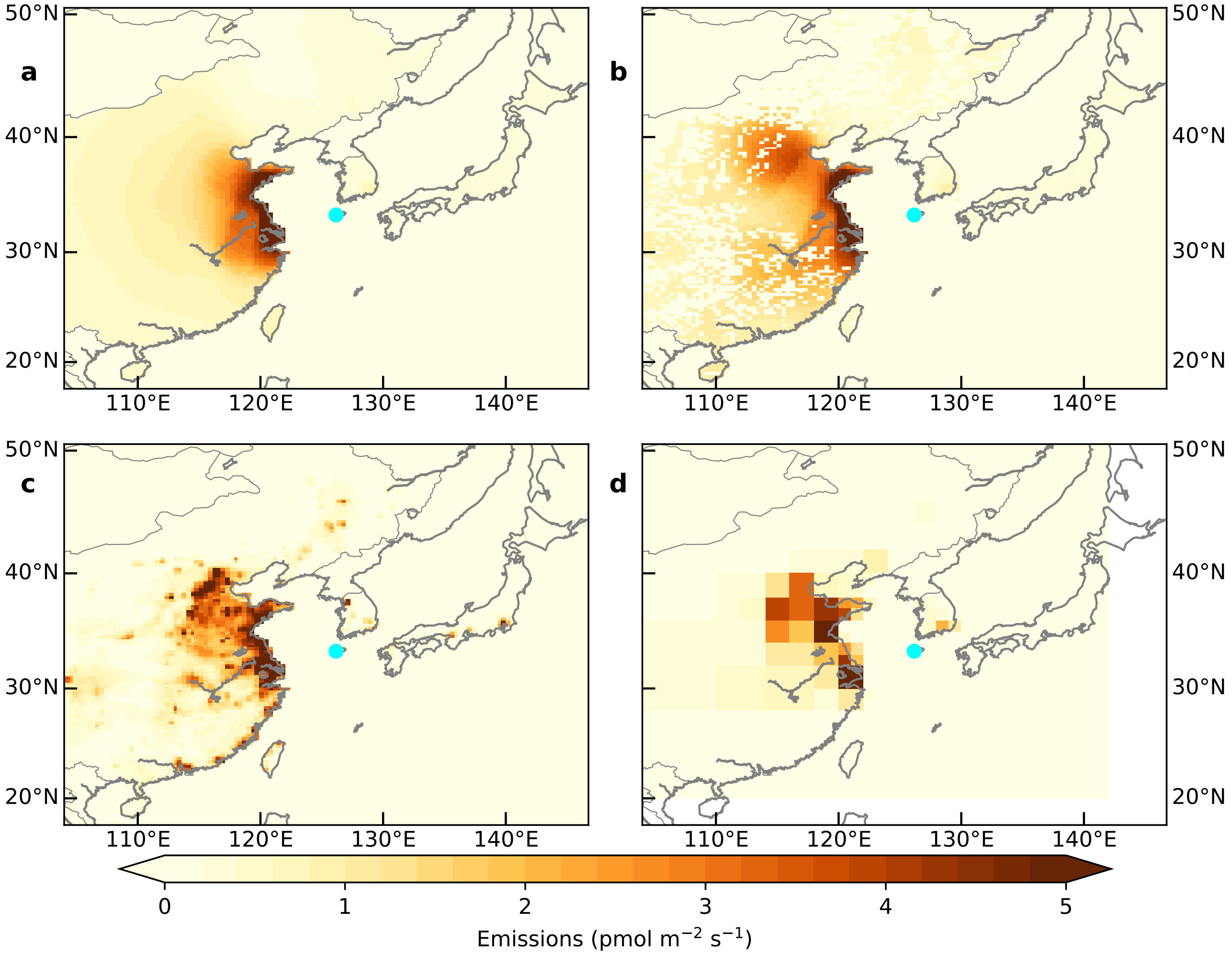

Figure S10. As Figure S9 but maps of the posterior distribution of emissions averaged over 2015–2016.
